# To robotize chemistry laboratories. An example of organic synthesis: 2-Boc-amino-N-hydroxy-3-phenyl-propionamide

**DOI:** 10.1155/S1463924602000032

**Published:** 2002

**Authors:** G. Christophe, L. Chapelant, S. Bostyn, C. Porte, A. Delacroix, H. D'Orchymont

**Affiliations:** 1 Equipe EA 21: Laboratoire de Chimie Industrielle du Conservatoire National des Arts et Métiers et Laboratoire de Productique chimique de l'IUT d'Orléans 2 rue Conté Paris Cedex 3 F-75141 France; 2 Synthélabo Biomoléculaire 16 rue d'Ankara Strasbourg Cedex F-67080 France

## Abstract

The paper describes the development of periodic modules used for the peptide synthesis of hydroxamic acid. A powder conveyor using the principle of positive weighing distribution is described. Purification is provided using automatic filtration and a liquid— liquid extraction module separation device. Device quality is improved using failure mode and effects analysis.


Journal of Automated Methods & Management in Chemistry
Vol. 24, No. 1 (January–February 2002) pp. 17–24

Taylor & Francis

Taylor & Francis Group

### To robotize chemistry laboratories. An example of organic synthesis: 2-Boc-amino-*N*-hydroxy-3-phenyl-propionamide

**G. Christophe[1], L. Chapelant[1], S. Bostyn[1], C. Porte[1]*, A. Delacroix[1] and H. d'Orchymont[2]**

[1]*Equipe EA 21: Laboratoire de Chimie Industrielle du Conservatoire National des Arts et Métiers et Laboratoire de Productique chimique de l'IUT d'Orléans, 2 rue Conté, F-75141 Paris Cedex 3, France*

[2]*Synthélabo Biomoléculaire, 16 rue d'Ankara, F-67080 Strasbourg Cedex, France.*

*The paper describes the development of periodic modules used for the peptide synthesis of hydroxamic acid. A powder conveyor using the principle of positive weighing distribution is described. Purification is provided using automatic filtration and a liquid–liquid extraction module separation device. Device quality is improved using failure mode and effects analysis.*

### Introduction

Research and development programmes are extremely important strategic aspects for international companies. The development of new products and improvements to qualities are important drivers for such companies. Because it is costly to develop new chemicals, management has placed a great deal of emphasis on high productivity and efficiency in new product creation. Optimum efficiency should provide an increase in productivity.

Automated systems are essential for several reasons but primarily to improve quality control and legislative control. Furthermore, the parameters within the experimental design must be controlled precisely.

Since the introduction of automated systems in productivity control some 15 years ago, the use of automation has spread rapidly to laboratories. The application of components for data processing and control of chemical analysis, more recently automation, has been used in experiments to develop new products. The so-called combinatorial chemistry should allow new products to be developed quickly and efficiently.

### Laboratory robotics

Currently, research is being applied to develop new modules and explore their chemical properties. Since the quantity of the product is smaller than that required for the development stage, reactors can now be miniaturized, i.e. only approximately 10 ml is required. However, it is difficult as well as prohibitively expensive to construct small reactors. A robot could replace research reactors. Some 15 years ago, Zymark developed and marketed a robotic arm with facilities to dilute and weigh samples [1]. Since then, the application of robotics in chemical laboratories has increased. Screening, developing and synthesizing new products is an area in which robotics has been successful.

Two approaches have been utilized in order to create molecular libraries: (*a*) versatile robot sites coupled to several devices and (*b*) synthesizers equipped with a Cartesian arm linked to basic modules including a pipetting station and a reaction block. The synthesizers are closed systems that carry out a limited number of operations, e.g. reagent additions, heating, cooling and filtration. These machines are convenient for high-throughout screening and have a proven capability for peptide system. However, they are less well adapted for liquid-phase synthesis.

Robotized sites have found preference by groups mechanizing the manual methods of reacting, thereby using the same equipment as far as possible [2]. The objective is to use the classified knowledge of organic chemistry to automate organic chemical reactions and to provide a high level of production throughput. To do this a versatile system is required and a robotized arm cannot achieve all the complex operations. Peri-robotic devices can be used to extend the usefulness of the robotized arms.

Commercial robotic systems are limited to basic operations. To develop fully automated system for the synthesis of hydroxamic acid, the industrial chemistry laboratory at CNAM has developed a range of peri-robotic modules [3]. These modules have been validated by the development of the synthesis described below.

### Example of a peptidic synthesis

The synthesis of 2-Boc-amino-*N*-hydroxy-3-phenyl-propionamide (where Boc is the tertiobutyloxycarbonyl) was chosen. It was classically applied in liquid-phase synthesis [4].

A heterogeneous mixture of 2-Boc-amino-3-phenyl-propionic acid (1.43 mmol), hydroxybenzotriazole hydrate (1.64 mmol) and dicyclohexylcarbodiimide (1.43 mmol) in methylene chloride was stirred at 0 °C for 45 min. Hydroxylamine hydrochloride (1.45 mmol) and triethylamine (1.73 mmol) were then added. The cooling bath was removed and the mixture stirred at room temperature overnight. The reaction mixture was diluted with ethyl acetate and dicyclohexylurea was removed by filtration. The ethyl acetate solution was washed with saturated potassium bicarbonate, 0.01 M hydrochloric

* To whom correspondence should be addressed. e-mail: cporte@cnam.fr solution and water-salted drying over magnesium sulphate and evaporation resulted in a white solid. Recrystallization in a mixture of chloroform and ethyl acetate gave a yield of 44%, the melting point being at 146 °C.

Initially, the chemist's manual recipe was translated into a grafcet (figure 1), which broke down the chemical process into a succession of unit laboratory operations.

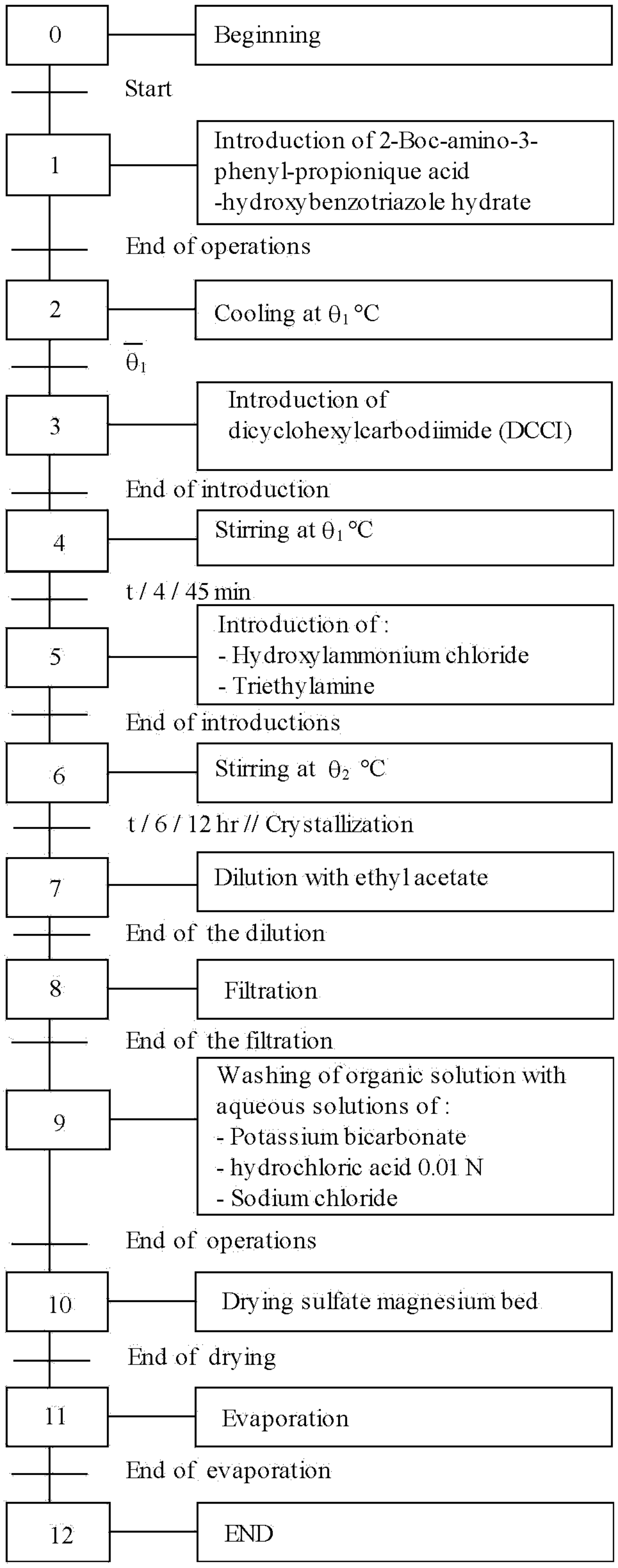

*Figure 1. First level grafcet.*

The robot had to manage the reactors displacements to reach the operations modules. Figure 2 describes this displacement for each step.

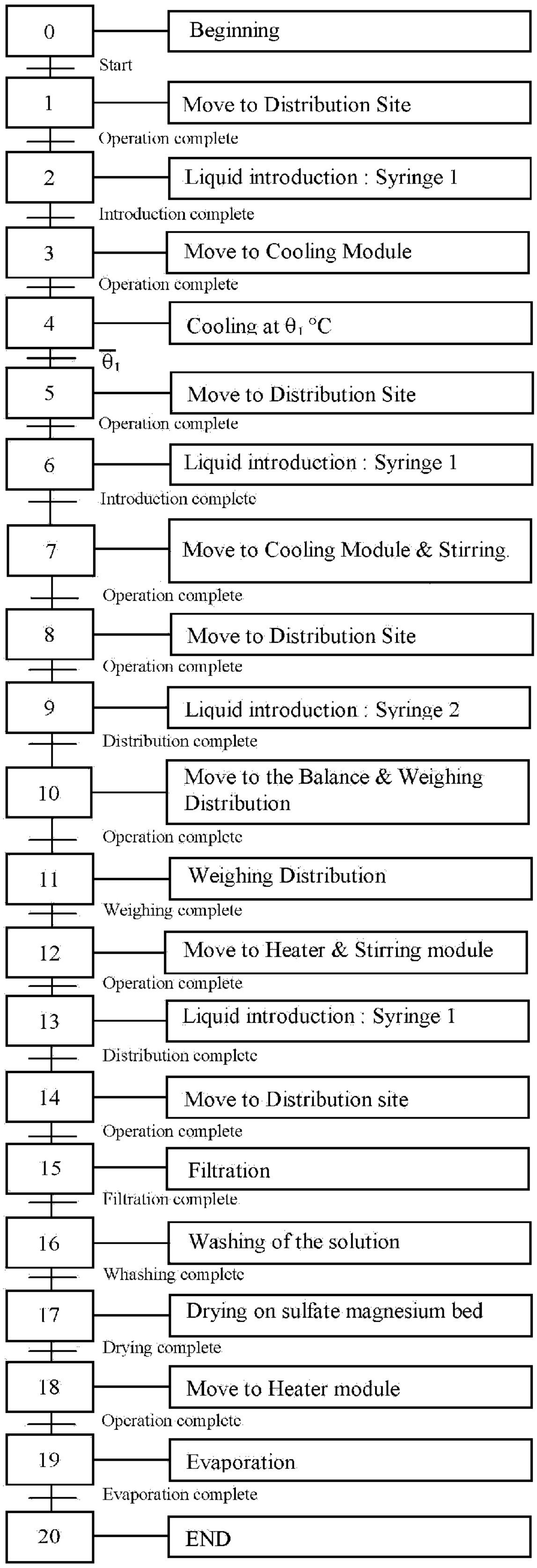

*Figure 2. Grafcet of the robot.*

During the introduction operations, the device's choice depended on the phase of the reactant. In the experimental procedure, most reactants were used in the solid form. The distribution difficulties of powder was due to an erratic coulability characteristic in relation to the particles' form, to their faculty to create electrostatic charges and to a pulverulent behaviour. No device really suited all applications.

To overcome the difficulties of introducing small quantities of solid, it was decided to prepare reactants solutions manually such as 2-Boc-amino-3-phenyl-propionic acid, 1-hydroxybenzotriazole hydrate (HOBT) and dicyclohexylcarbodiimide (DCCI). Since the hydroxylammonium chloride was not very soluble in methylene chloride or DMSO at ambient temperatures, it could only be introduced in its solid form, thus requiring the use of a powder.

The cooling module is made up of a thermocryostat (Bioblock, France) equipped with a specific reactor block. The heating–stirring and evaporation module was from Pierce (France). The weighing module is an electronic balance (Model AE 200) from Mettler (France).

Each operation involving solid reactants, such as purification or filtration, was difficult to control and required a long manual and cumbersome intervention. Since it was desirable not to restrict automation to liquid transfers, a laboratory powder conveyor and a purification module were developed. The latter involved a filtration, and a liquid–liquid extraction was developed.

### Laboratory powder conveyor

The powder conveyor using the principle of positive weighing distribution is shown in figure 3. The conveyor, placed on a ball bearings rail (I), was moved by a pneumatic jack (J) to disengage it outside the balance. Its position was confirmed with a sensor (K).

The synthesis reactor in which the powder must be distributed was on the balance tray. The device was constituted of a geared motor driven screw (B). It was fed with powder by a hopper (C) equipped with a loosening tool (D) and an electrovibrator (E), which eliminated the risks of ark or chimney formation. The hopper lies on a rail (F), which is removed easily and allows easy change of the type of screw. The conveyor was set on a mobile tray (G), which allowed the introduction of the device into the balance above the tray. The distance between the exit of the screw and the reactor was reduced to a minimum, so avoiding the loss of product during introduction of the powder.

An antistatic bar (not shown) was installed inside the balance to eliminate any electrostatic charges and the flying particles. A muff (H), opened on the antistatic bar side, avoided the scattering of solid particles.

### Purification module

### *Filtration of the solution*

The principle selected was that which was employed classically by the chemists, i.e. a filtration by aspiration. The filtration system includes a removable filter.

The reactor is at the liquid distribution site. A sample nozzle driven by a pneumatic jack enters the reactor to bring the sample (figure 4). The vacuum setting of the high part of the filter (maintained by the vertical pressure of a second jack) aspirates the reactor solution. Ethyl acetate was added from an automatic syringe to precipitate the dicyclohexylurea (DCU). The solution was then filtered by the vacuum created in the low filter to eliminate the DCU fine crystals. The filtrate was introduced directly in the washing device by a vacuum. The empty reactor and all appliances used during the operation were rinsed with ethyl acetate.

The filtration module was equipped with a pneumatic jack (not shown), which allowed the filter to be exchanged to avoid cross-contamination between samples.

### *Washing of the solution*

The purification operation consists of a discontinuous liquid–liquid extraction module. The major difficulty of this operation was the detection of the interface between the two liquid phases.

The liquid–liquid extraction module must operate reliably whatever the liquid–liquid interface. The chemists, during manual liquid–liquid extraction, had used a modified vessel; spherical separating funnels permitted the mixing of the two liquid phases and allowed the precise detection of the interface during the separation phase. The automated system followed the manual procedure closely.

Figure 5 shows the separation device consisting of a miniaturized spherical separating funnel and a series of solenoid valves to introduce the different washing liquids and separate the phases.

The organic solution (after the filtration module) was introduced using a vacuum pipette. The first washing solution was injected in the vial by an automatic syringe. The reagents were mixed using a flow of compressed air. After 1 min of agitation, the mixture was decanted by the application of an atmospheric pressure of air in the vial (3 min). The solution was then emptied. A capacitive detector was used to detect the interface and then directed the solutions in distinct tanks. The aqueous phase was oriented toward waste. The organic phase was directed towards an intermediate storage reservoir. It was finally pumped again in the vial, ready for a new washing operation.

After the last washing, the organic phase, contained in the storage reservoir, was transferred directly to the drying module.

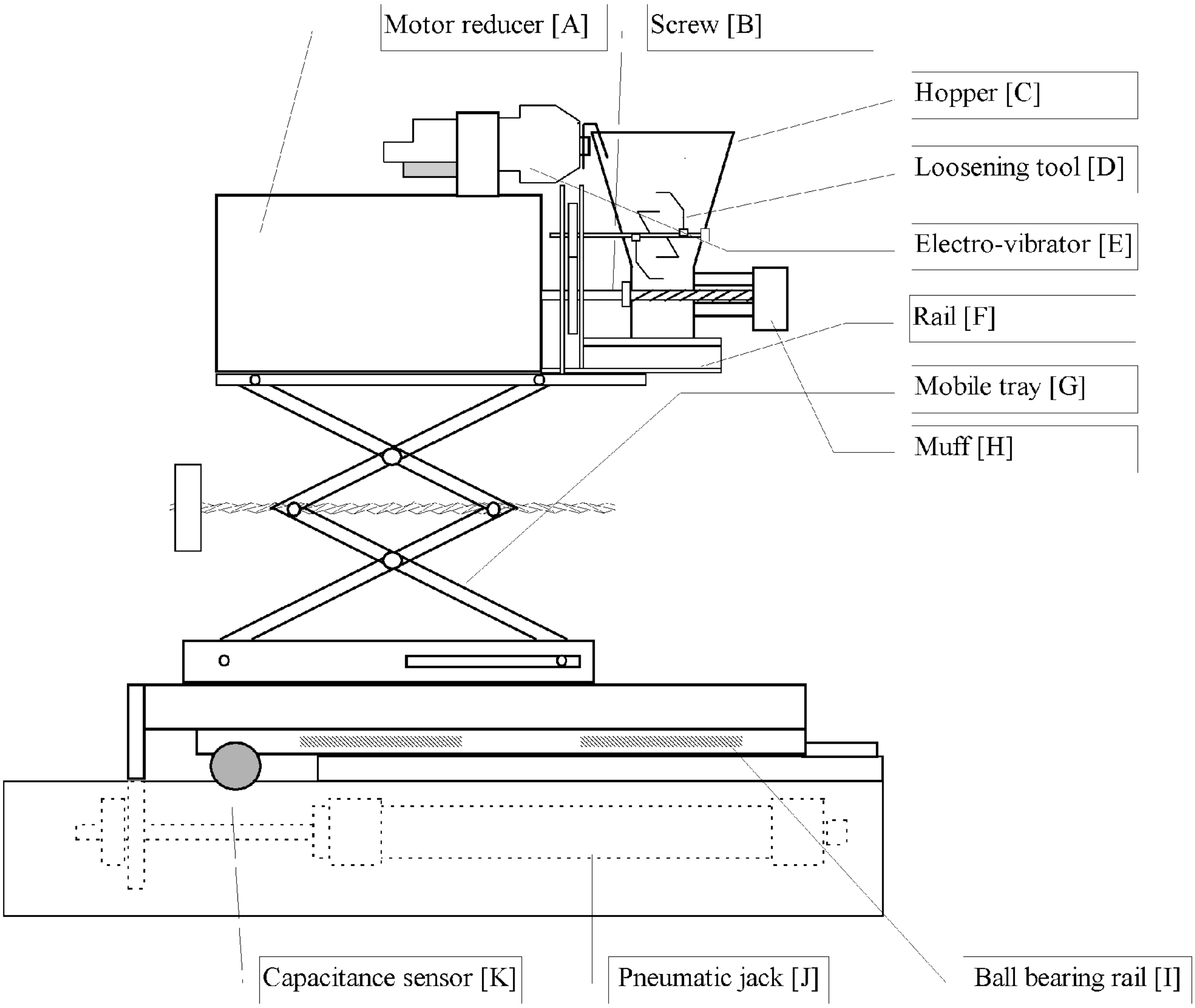

*Figure 3. Laboratory conveyor of pulverulent powder.*

*Drying of the solution*

This module (figure 6) consisted of a bed of magnesium sulphate laid on a filter into a cylindrical container. The organic phase was introduced into the container from the storage reservoir of liquid–liquid extraction. An air stream pushed it across the bed; a tube into the bed has limited the pressure. The organic phase was then collected in a reactor (20 ml) designed for evaporation, brought previously by the robot. The magnesium sulphate bed in the filtration module was changed using a jack system.

### Reliability study

In the analysis or chemical synthesis, the use of robotics requires the insurance of good appliance reliability. The realization of a fully robotized synthesis included many operations that requires a high precision of movement, such as capping and uncapping the reactors, the reagent bottle, etc. The more complex the automated system, the greater the risk that an operation failure might occur which would stop the synthesis sequence. A sample system offers the best solution.

To avoid frequent operator interventions, the system must be as reliable as possible. The authors therefore studied failure mode and effect analysis to evaluate, classify and compensate for the robotized systems failings.

A large number of methods and techniques were relevant for this research [5]. The two most commonly used were: (*a*) failure mode and effect analysis, and (*b*) the failure cause tree. The first method was chosen since it enabled the authors to verify that no failure led to the failure of a task as a whole [6]. In addition, it allowed a step-by-step approach to the analysis. Moreover, the failure mode and effect analysis was, by its nature, adapted to the survey of delimited subsets, where a large number of little interacting causes could exist. Indeed, the system comprised a multiplicity of independent devices. Most have an elementary functioning mode that limits the risk of failure of each device.

This failure mode analysis is an inductive method used in a system security study, which identifies all the failure modes, having an effect on the system [7]. Its

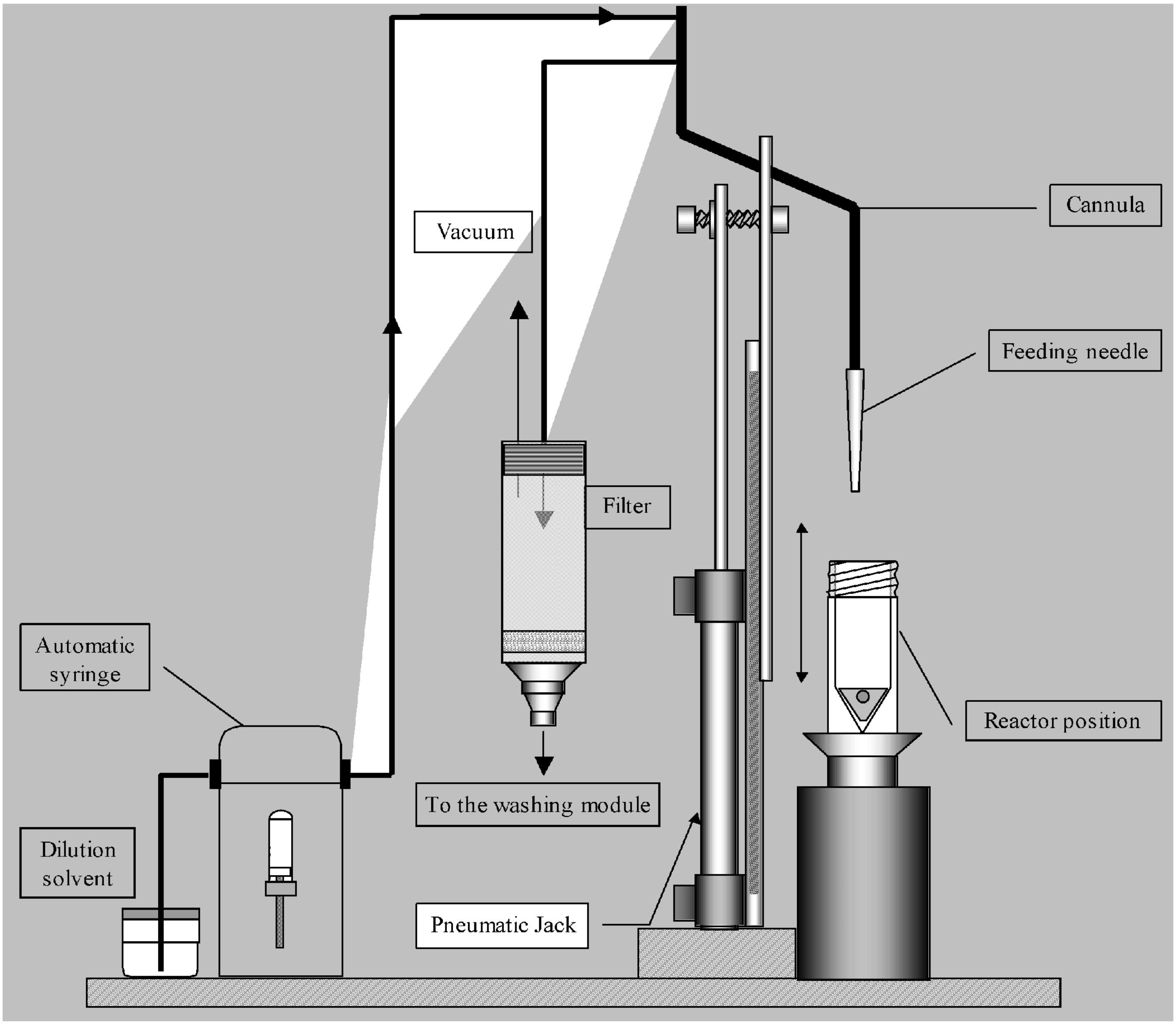

*Figure 4. Filtration device.*

goal was, first, to provide an exhaustive research of all the events, which could be responsible for the synthesis failure. Second, the ordering and examination of these enabled the improvement of the availability of the system for use.

The failure mode and effect analysis aimed at filling a table in which each elementary failing cause occupied a line (tables 1 and 2).

The robotized site was considered as a whole: data processing, the articulated arm and all the peri-robotic devices. Each previous element, taken individually, was a subsystem and possible locus of one or several failings. Only material failings were taken into account, the human failings being reduced in a completely automated system such as the robotized site.

In the example (table 3), consideration was given to the system of weighing (registered in column [A]). In the following column, [B] failures were registered for the operation of weighing. Column [C] corresponded to one or several causes to each of these modes of failure.

Local effects of this failure (column [D]) were then identified and entered in the set of the system (column

*Table 1. Consequences of a failing and level of gravity.*

| *Consequences of the failing* | *Level* | |
|---|---|---|
| No damage, either on the system or on the current syntheses | 1 | unimportant |
| Admit palliative or correctives such as there is no damage either on the system or on the current synthesis | 2 | rare |
| Requires taking immediate action with loss of the synthesis of the current reactor, continous system process | 3 | seriousness |
| Do not admit palliatives or correctives with loss of totality of the current syntheses | 4 | major |

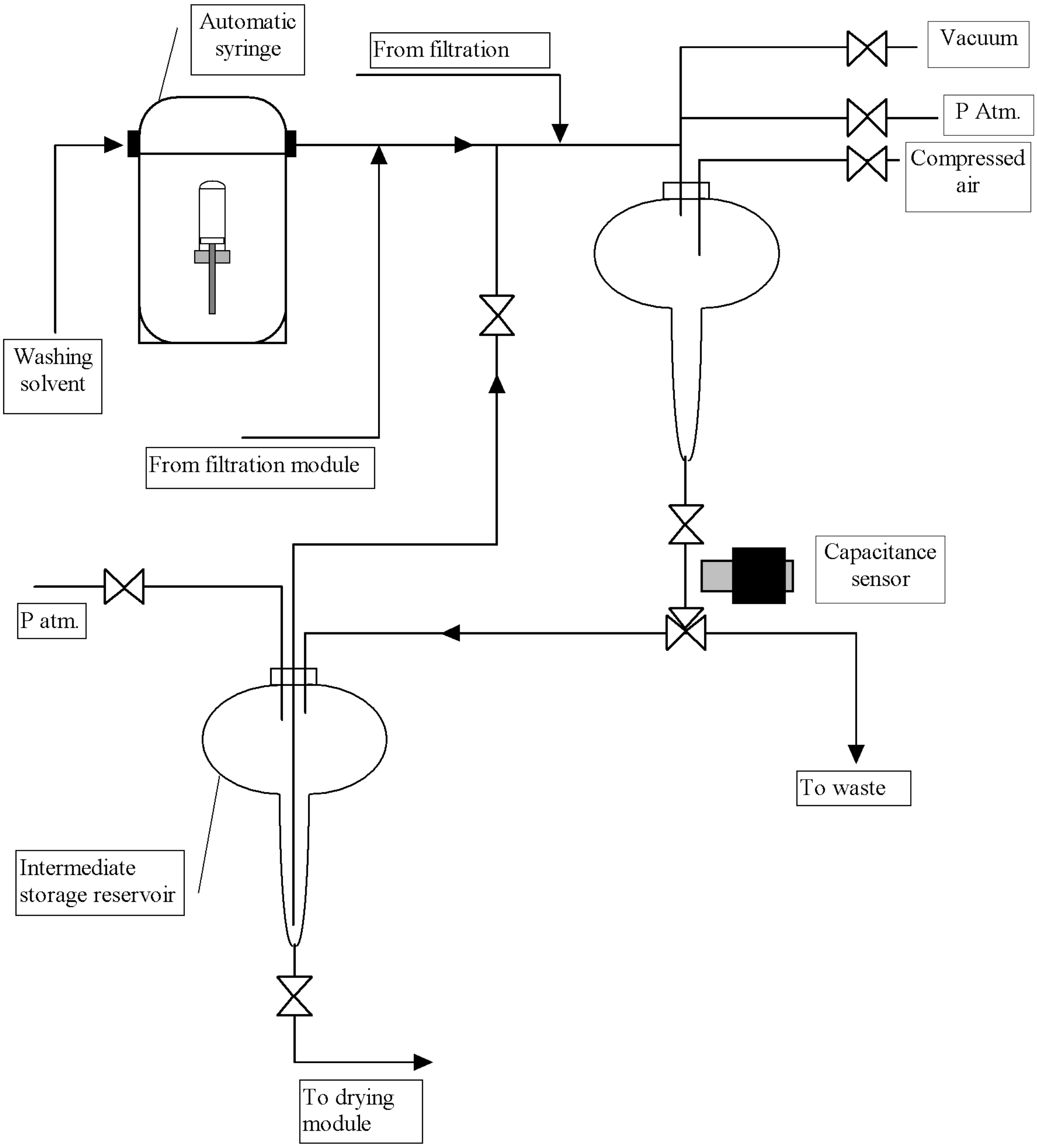

*Figure 5. Liquid–liquid separation device.*

*Table 2. Frequencies of a failing and level of probability.*

| *Frequency of the failing* | *Level* | |
|---|---|---|
| Probability of very weak failing | 1 | very rare |
| Weak probability of failing | 2 | rare |
| Possibility of failing | 3 | possible |
| Great probability of failing | 4 | frequent |
| Very great probability of failing | 5 | very frequent |

[E]). Existence and functioning of any compensation available were noted in column [F].

The gravity of events was classified traditionally in six categories. These were classification to the robotic site, and four are valued (table 1).

Indeed, upper levels (5 and 6) in the classic method were reserved for severe failings that affect the scale of the establishment. For this study, the most severe was resumed for the complete event loss of the reactors (no. 4) (table 2).

The severity of events was noted in column [G], while their probability of appearance was noted in column [H]. The approach of these two figures (columns [G] and [H]) allowed us to classify risks (column [I]) (table 3).

When the system was complex, the numbering of risks assisted greatly in their recognition. It was then possible to note these numbers on a probability diagram for severity and to recognize the most destructive ones and provide strategies to overcome them.

This method was a great help in the recognition of the tasks to study and allowed the authors to reduce the

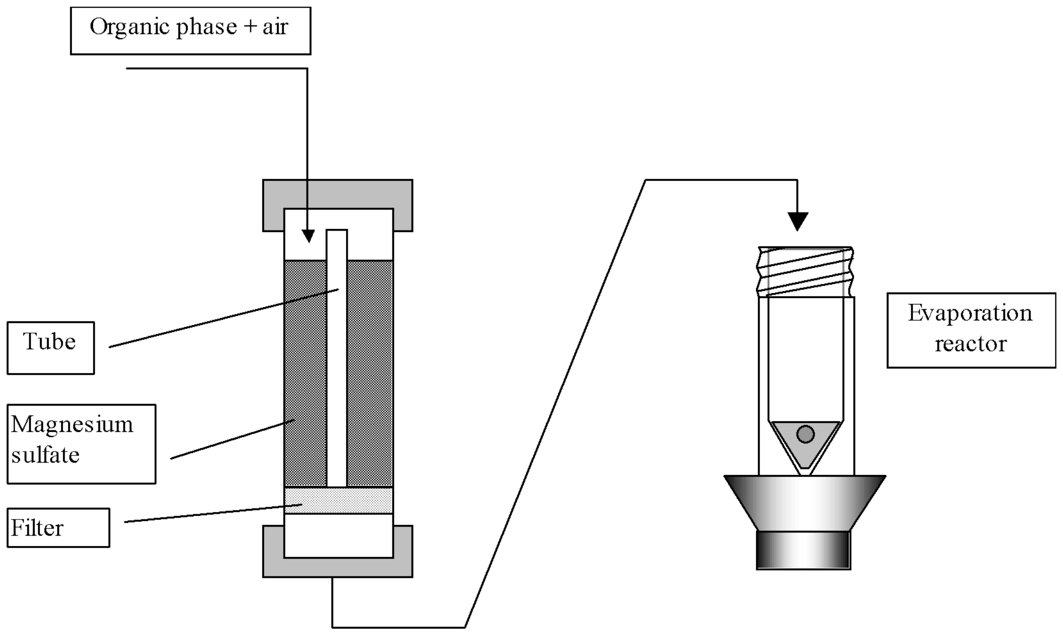

*Figure 6. Drying on bed device.*

*Table 3. Example of a failure mode and effect analysis.*

| *Composant–assemblages–by-system* | | | *Failure effect* | | | | | |
|---|---|---|---|---|---|---|---|---|
| *Name–references–function* | *Failure mode* | *Causes* | *Local* | *On the system* | *Existence of compensations* | *Gravity* | *Probability* | *Risk* |
| Balance | reactor is not detected | great error on robot position | reactor is falled over in the balance | stopping of the robot program | none | 4 | 1 | 41 |
| | | gap on the peripheric position | *idem* | *idem* | none | 4 | 1 | 41 |
| | default on RS232 communication | unknown | program is not beginning | none | none | 1 | 3 | 13 |
| [A] | [B] | [C] | [D] | [E] | [F] | [G] | [H] | [I] |

system failings, often in a very simple way. The use of such a study, and its continuation, was indispensable in order to analyse the availability and the throughput of a robotized station.

### Conclusion

The automated synthesis yielded products with satisfactory purities (2-Boc-amino-*N*-hydroxy-3-phenyl-propionamide) with a satisfactory yield (66%). On the six experiments conducted, the reproductivity of results was about 15%. Quantities produced for a reactor ranged from 50 to 80 mg.

However, at this scale, efficiency is significantly lower than that observed manually, normally about 98%. It may be attributed to the loss of product in volumes transferred from the module of purification.

The difference of purity of products can be explained by the non-replacement of the filter, which seals in DCU. Furthermore, reactions are not carried out in strictly anhydrous conditions. During the introduction of the reagents, a partial hydration of the reaction environment was possible.

This tool is not limited to the analysis, screening or synthesis of small quantities. However, its present cost is too high for some chemistry fields. Its economic justification revolves around the repeatability of the tasks and the existence of effective and efficient automated devices.

Nevertheless, the recent robotized site multiplication shows the viability of the unit. Furthermore, the development of more varied peri-robotic devices will give rise to a larger utilization. Finally, the data processing, essential for data storage, management and data operating allows for the use of supervisor programs and expert systems to help the mundane tasks of the analytical chemists.

### References

1. Zenie, F., A trio of new ventures. *Analytical Chemistry*, **56**, 1984, 256A.
2. Bostyn, S. La robotique: un nouveau moyen pour l'étude des synthèses chimiques à l'échelle du laboratoire de recherche. Etude et mise au point d'un site robotique. Doctoral thesis, l'Université of Pierre et Marie Curie Paris VI et Conservatoire National des Arts et Métiers, 1993.
3. Khoulif, Z., Analyse chimique en ligne à l'échelle initiale. Etude et mise au point d'un site robotique. Doctoral thesis, l'Université Pierre et Marie Curie Paris VI et Conservatoire National des Arts et Métiers, 1995.
4. Chapelant L., La robotique de laboratoire: nouveaux modules périrobotiques. Application à la synthèse peptidique du 2-boc amino-N-hydroxy-3-phenyl-propianamide. Doctoral thesis, l'Université Pierre et Marie Curie Paris VI et Conservatoire National des Arts et Métiers, 1997.
5. UIC, Les différentes méthodes d'analyse de sécurité dans la conception d'une installation chimique. *Les cahiers de sécurité*, n° 3 and 4, 1981.
6. Diallo, M. B., Etude de sécurité et mise en œuvre d'une architecture de gestion de commande automatisée d'un pilote chimique polyvalent et automatisé. Doctoral thesis, l'Université Pierre et Marie Curie et du Conservatoire National des Arts et Métiers, 1994.
7. Villemeur, A., Sûreté de fonctionnement des systèmes industriels, Collection de la Direction des études et recherches d'Electricité de France, Eyrolles, Paris, 1988.

